# An update on environment-induced pulmonary edema – “When the lungs leak under water and in thin air”

**DOI:** 10.3389/fphys.2022.1007316

**Published:** 2022-10-07

**Authors:** Kay Tetzlaff, Erik R. Swenson, Peter Bärtsch

**Affiliations:** ^1^ Medical Clinic, Department of Sports Medicine, University of Tübingen, Tübingen, Germany; ^2^ Department of Medicine, University of Washington, Seattle, WA, United States; ^3^ Division of Pulmonary Medicine and Critical Care, University of Washington, Seattle, WA, United States; ^4^ Department of Internal Medicine, University of Heidelberg, Heidelberg, Germany

**Keywords:** pulmonary edema, immersion, altitude, swimming, pulmonary artery pressure

## Abstract

Acute pulmonary edema is a serious condition that may occur as a result of increased hydrostatic forces within the lung microvasculature or increased microvascular permeability. Heart failure or other cardiac or renal disease are common causes of cardiogenic pulmonary edema. However, pulmonary edema may even occur in young and healthy individuals when exposed to extreme environments, such as immersion in water or at high altitude. Immersion pulmonary edema (IPE) and high-altitude pulmonary edema (HAPE) share some morphological and clinical characteristics; however, their underlying mechanisms may be different. An emerging understanding of IPE indicates that an increase in pulmonary artery and capillary pressures caused by substantial redistribution of venous blood from the extremities to the chest, in combination with stimuli aggravating the effects of water immersion, such as exercise and cold temperature, play an important role, distinct from hypoxia-induced vasoconstriction in high altitude pulmonary edema. This review aims at a current perspective on both IPE and HAPE, providing a comparative view of clinical presentation and pathophysiology. A particular emphasis will be on recent advances in understanding of the pathophysiology and occurrence of IPE with a future perspective on remaining research needs.

## Introduction

Acute pulmonary edema is a potentially life-threatening condition that is caused by accumulation of extravascular fluid in the lungs. Traditionally, two fundamentally different types of pulmonary edema have been described in humans: cardiogenic pulmonary edema (also referred to as hydrostatic pulmonary edema), caused by an increase in pulmonary capillary hydrostatic pressure secondary to left heart failure, and non-cardiogenic pulmonary edema which develops following injury to the alveolo-capillary barrier (also referred to as permeability pulmonary edema), often being associated with acute respiratory distress syndrome (Ware and Matthay, 2005).

However, pulmonary edema may even occur in young and healthy individuals when exposed to extreme environments, such as immersion in water, or at high altitude. Immersion pulmonary edema (IPE) was first described in self-contained underwater breathing apparatus (scuba) divers who suffered from dyspnea attributable to pulmonary edema after open sea dives in cold water (Wilmshurst et al., 1989). Initially reported as diving-induced pulmonary edema, subsequent reports highlighted the occurrence of IPE in military trainees (Weiler-Ravell et al., 1995), triathletes (Carter and Koehle, 2011), swimmers (Pons et al., 1995), breath-hold divers (Boussuges et al., 1999), or even aqua-joggers (Wenger and Russi, 2007). An emerging understanding of IPE indicates that an increase in pulmonary artery and capillary pressures caused by a substantial redistribution of venous blood from the extremities to the chest, in combination with stimuli amplifying the effects of water immersion such as exercise, ambient cold, constrictive wetsuits, and negative pressure breathing.

While there are commonalities in clinical presentation between IPE and HAPE, the latter is thought to be caused by leakage of the alveolar-capillary barrier secondary to uneven hypoxic pulmonary vasoconstriction (Bärtsch and Swenson, 2013). This review aims at a current perspective on both IPE and HAPE, providing a comparative view of clinical presentation and pathophysiology, and highlighting recent advances in understanding of the pathophysiology.

### Clinical presentation of IPE

Clinical presentation of swimming-induced IPE is characterized by an acute onset of nonspecific symptoms of breathlessness and cough in 70–80% of cases, hemoptysis (68%) with chest tightness or pain reported less frequently (Grünig et al., 2017). Slade et al. (2001) reported a series of eight cases of IPE while scuba-diving. Dyspnea and hemoptysis were prevailing symptoms in these subjects, followed by cough and fatigue, with an onset of symptoms during the dive or when surfacing.

**FIGURE 1:**
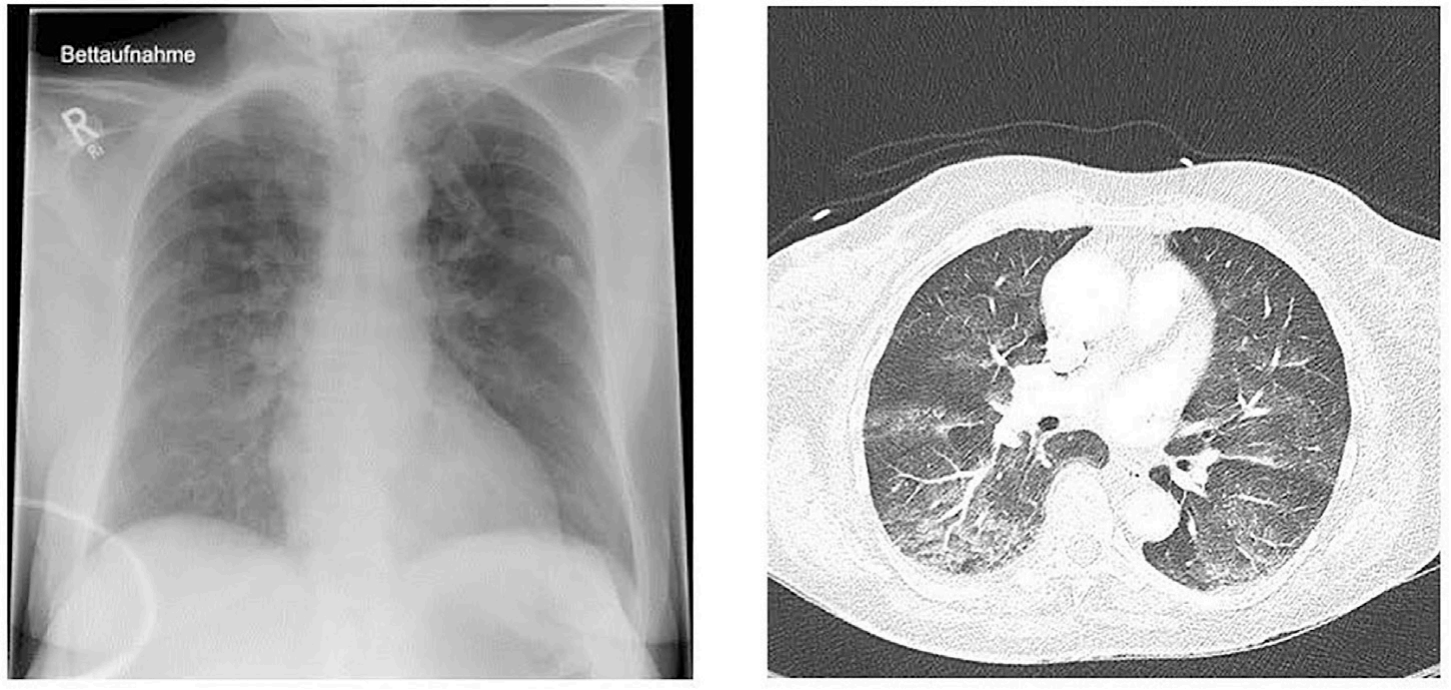
Chest x-ray (left) and computed tomography (right) of a 72-year-old previously healthy and physically fit female after swimming in a cold lake (Courtesy of Karin Hasmiller, MD, Berufsgenossenschaftliche Unfallklinik, Murnau, Germany).

Radiographs and computerized tomography scans of the lungs may demonstrate infiltrates and patchy acinar and lobular ground-glass and consolidative opacities (Figure 1). However, presentation of IPE may be unilateral (Hårdstedt et al., 2020), and has been reported in military trainees swimming in a lateral decubitus position with the radiographic findings in the dependent submersed lung (Mahon et al., 2002).

While there is some variability in reported incidence of IPE that may be explained by different settings of IPE, nature of data collection, and a high rate of spontaneously resolving cases, it is obvious from recent large and prospective cohort data that IPE occurs more frequently than previously thought, especially in elderly subjects (Hårdstedt et al., 2022). Highest incidences of 5% or more are reported in military trainees during strenuous swims (Weiler-Ravell et al., 1995; Volk et al., 2021).

### Clinical presentation of HAPE

**FIGURE 2:**
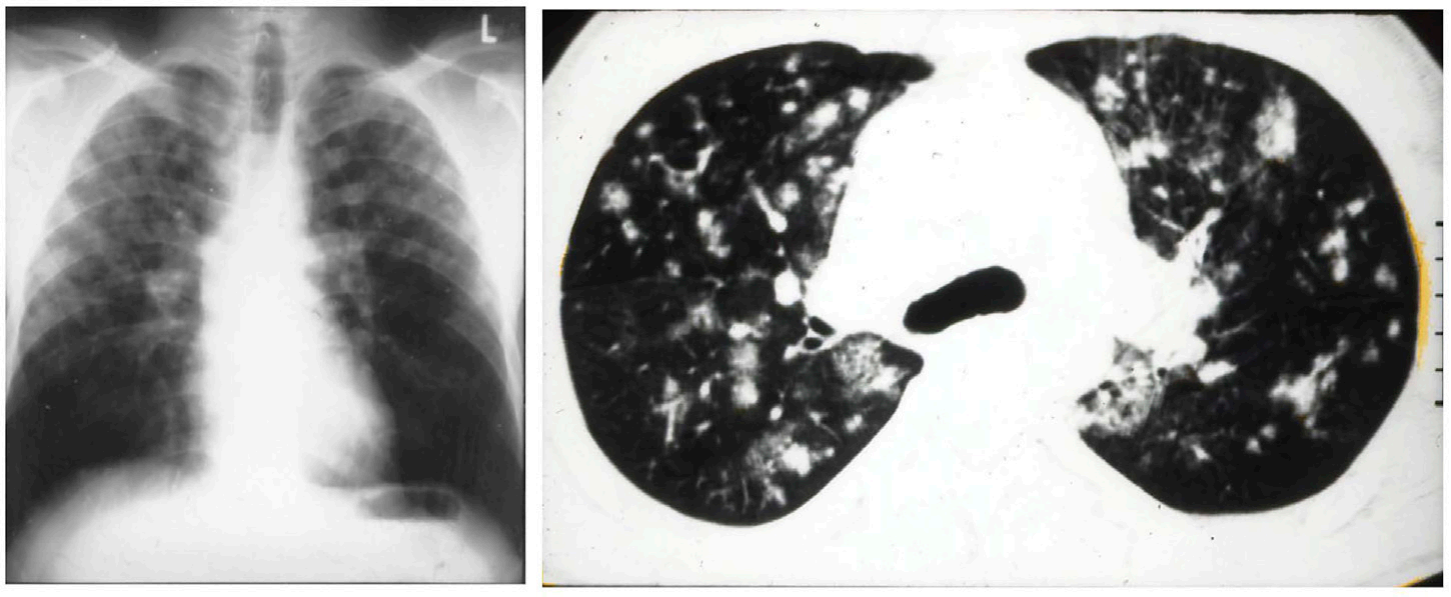
Figure 1 **(A)** radiograph of a 37-years-old male mountaineer with high-altitude pulmonary edema (HAPE) that shows a patchy to confluent distribution of edema, predominantly on the right side. **(B)** computerized tomography scan of 27-year-old mountaineer with recurrent HAPE showing patchy distribution of edema. Taken from Bärtsch, P, Mairbäurl, H., Maggiorini, M., Swenson, E. 2005. Physiological aspects of high-altitude pulmonary edema. J Appl Physiol, 98, 1101–1110.

HAPE is a non-cardiogenic pulmonary edema that may occur in healthy and often young individuals exposed to high altitude. Early symptoms are inappropriately greater dyspnea with exercise, dry cough and loss of performance. More advanced cases present with dyspnea at rest, cyanosis, rales and pink, frothy sputum (Bärtsch and Swenson, 2013). In severe cases arterial PO_2_ may be as low as 19 mmHg and arterial oxygen saturation below 40% at altitudes of 4,500–5,000 m (Bärtsch et al., 1987). These observations can explain the estimated mortality of about 50% in HAPE without appropriate treatment, while symptoms - except for reduced exercise tolerance - disappear within hours after rapid descent to low altitude. Radiographs and computerized tomography scans of the lungs demonstrate patchy distribution of the edema (Figure 2), which resolve completely within 3–5 days (Vock et al., 1991).

HAPE usually develops over 1–2 days at altitudes above 3,000 m. The risk increases with altitude and fast ascent. The prevalence in trekkers ascending within 6 days to 5,400 m is about 1–2% (Hackett et al., 1976). When ascending to 4,559 m within 24 h, the prevalence increases in mountaineers without a history of HAPE to 6% and in those with a history of HAPE to 62% (Bärtsch et al., 2002). The latter data show that there are individuals, who are particularly susceptible to HAPE.

### Pathophysiology of IPE

Recent advances in understanding the pathophysiology and occurrence of IPE indicate that IPE is the result of the interplay between individual predisposition and environmental circumstances, including equipment used in the case of scuba divers. The central contributing factor, by definition, is water immersion to any depth, leading to raised central blood volume and hence cardiac filling pressures. Both, physical exercise and ambient cold will augment the cardiovascular effects of immersion, however, other factors may contribute to fluid extravasation into the lung, such as compression of the thorax by wetsuit, negative pressure breathing etc.

Passive and active redistribution of venous blood from the extremities and viscera to the heart and pulmonary vessels will increase pulmonary artery pressure. Cold-induced sympathetic nervous activation causes constriction of capacitance veins, which account for 50–60% of total blood volume. Exercise will further increase thoracic blood volume and left atrial, pulmonary artery, and capillary pressures. Cold-water immersion and exercise both increase mean arterial, pulmonary arterial, and pulmonary artery wedge pressures during prone immersion having additive effects (Wester et al., 2009). “Head only” thermal protection to eliminate cold-induced reflexes from scalp and facial sensory nerves did not attenuate the rise in pulmonary artery pressure. Peacher et al. (2010) investigated cardiopulmonary dynamics in 10 subjects at rest and during 16 min of exercise submersed at 1 atm absolute breathing air and at 4.7 ATA in normoxia and hyperoxia. Mean pulmonary artery pressure varied widely between individuals (up to a 2–3-fold difference among subjects), indicating the possibility of differential susceptibility to IPE among individuals, as has been shown more definitively for HAPE and pulmonary vascular pressures. There were measurements of mean pulmonary artery pressure during submaximal exercise that rose above the threshold for pathological values which was in apparent contrast to dry exercise, in which such high values are only observed during maximal exercise (Kovacs et al., 2009). Breathing a hyperoxic gas mixture during exercise in cold water did not protect against pulmonary hypertension consistent with enhanced peripheral vasoconstriction and its effects on cardiac afterload and preload (Fraser et al., 2011).

These observations support elevated pulmonary vascular pressures as the cause of IPE, however, do not prove that susceptible individuals may have an excessive rise in pulmonary artery pressure caused by exaggerated peripheral venoconstriction or nonhomogeneous pulmonary vasoconstriction, as has been suggested for HAPE. Moon et al. (2016) investigated pulmonary and systemic hemodynamics, expired gas, and blood gases in 10 subjects at supine dry rest (baseline), after submersion in cold water, and during moderate cycle ergometer exercise while submersed. These subjects had a history of at least one previous episode of IPE and underwent cardiovascular screening to exclude cardiovascular disease. During submersed exercise mean pulmonary artery and pulmonary artery wedge pressures increased despite lower cardiac output in comparison with a historical control group of non-susceptible healthy subjects. The phosphodiesterase-5 antagonist sildenafil reduced pulmonary vascular pressures and pulmonary vascular resistance but had no effect on other hemodynamic variables (Moon et al., 2016). PDE-5 inhibitors are known to dilate venous capacitance vessels and by this action will reduce the redistribution of blood to the thorax (Ng and Pang, 1998; Inoue et al., 2001). These data strongly suggest a pulmonary vasoconstrictive component and peripheral venoconstriction that is alleviated by sildenafil in line by its well-characterized vascular smooth muscle relaxation effects in all circulatory beds.

Some of those individuals who showed an immediate increase in PAP when submersed, however, developed mitral regurgitation that was not clinically apparent under dry conditions (Beck et al., 2022). Thus, subclinical cardiac disease that manifests during immersion only may account for IPE in certain individuals. This would be consistent with the observation that increased age significantly increases the odds of suffering from IPE during strenuous swimming (Hårdstedt et al., 2022) (Table 1).

**TABLE 1:** Risk factors for IPE (S_a_O_2_ = oxygen saturation, PFT = pulmonary function test, FVC = forced vital capacity, FEV_1_ = forced expiratory volume in 1 s).

Study	Design	Cohort	Investigations	Risk factor
Wilmshurst et al. (1989)	Controlled prospective cohort	11 divers with history of IPE	Forearm blood flow and blood pressure during exercise and cold exposure	Abnormal vascular resistance during cold exposure
Weiler-Ravell et al. (1995)	Prospective cohort	30 military trainees	S_a_O_2_, history of drinking several liters before swim	Severe exertion. overhydration
Shupak et al. (2000)	Prospective cohort	35 military trainees	S_a_O_2_, symptoms, auscultation, PFT	Significantly lower pre-swim FVC and FEV_1_ in eight subjects with severe IPE
Miller et al. (2010)	Online survey and case-control study	31/1,400 responders among US triathletes	Patient reported symptoms, multivariate analysis	History of hypertension; fish oil consumption; female gender; course distance
Peacher et al. (2015)	Observational cohort and literature review	36 subjects (10 inpatients) and 292 reviewed cases	Symptoms, medical history	Cardiopulmonary disease and other comorbidities in 75 and 25.9% of reviewed cases
Moon et al. (2016)	Controlled clinical trial	10 subjects (divers and triathletes) with a history of IPE and 20 controls	Cycle ergometer exercise while submersed in 20°C water	Significantly greater mean PAP and wedge pressure in IPE subjects, no difference after sildenafil intake
Boussuges et al. (2017)	Observational cohort	106 French Navy divers performing 263 dives	Extravascular lung water determined by ultrasound	Exercise intensity (Borg-scale)
Hårdstedt et al. (2022)	Prospective cohort	45,913 recreational/competitive swimmers over four consecutive years	IPE diagnosis based on lung ultrasound, clinical findings	Age (odds 12.74 in >61 years compared to 18–30 years); Female gender (age-adj. odds 8.59 compared to men)
Beck et al. (2022)	Case series	Four subjects with history of IPE	Echocardiography during head-out immersion	Subclinical mitral valve regurgitation

### Pathophysiology of HAPE

**FIGURE 3:**
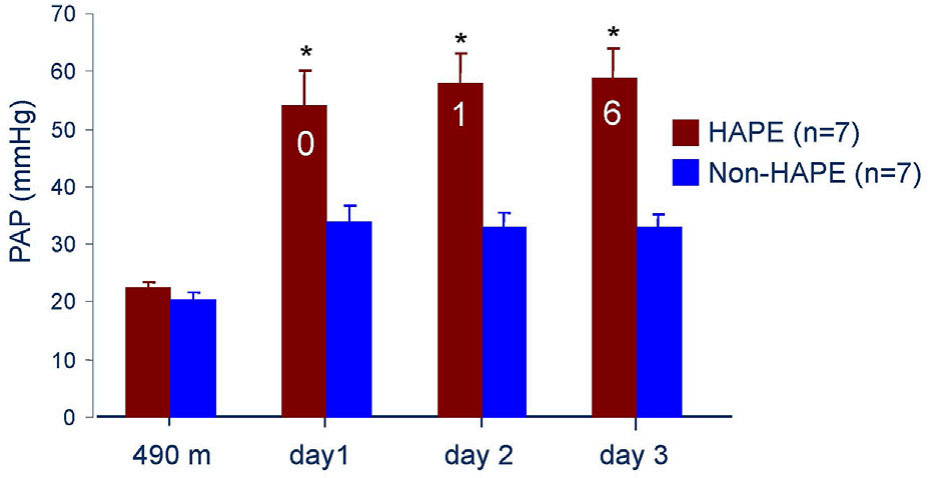
Systolic pulmonary artery pressure at 490 m and 4,559 m in seven subjects developing HAPE (red bars) and seven control subjects (blue bars). The numbers in the red bars indicate the subjects with HAPE at a particular examination. Data taken from Bärtsch P. et al., 1991. Prevention of high-altitude pulmonary edema by nifedipine. N Engl J Med 325: 1284–1289.

**FIGURE 4:**
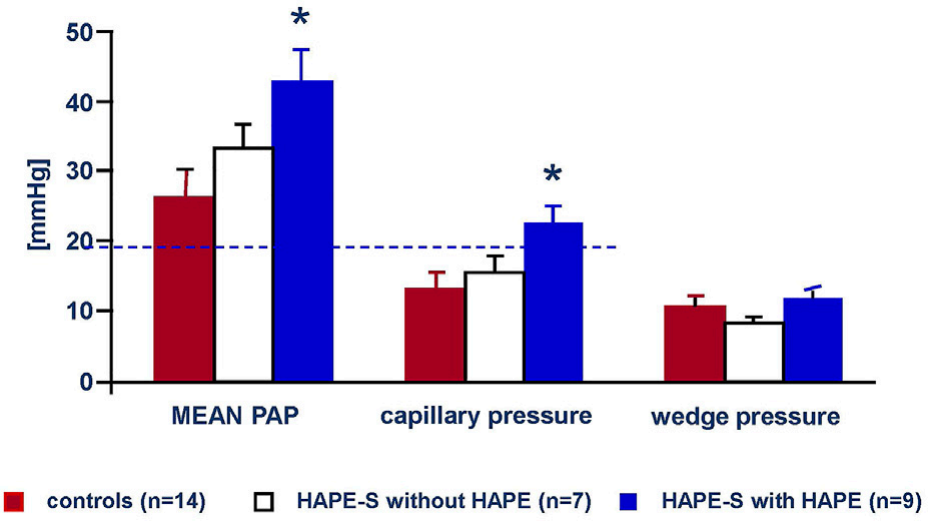
Mean arterial, capillary and Wedge pressures of the pulmonary circulation on the second day at 4,559 m in 14 control subjects (red bars), seven HAPE susceptible individuals with no HAPE during a 3 days exposure (white bars) and nine HAPE susceptible subjects with HAPE during a 3 days exposure at 4,559 m. Data taken from Maggiorini et al., 2001. High-altitude pulmonary edema is initially caused by an increase in capillary pressure. Circulation 103: 2078–2803.

Hypoxia at high altitude causes hypoxemia, which increases with altitude. At 4,559 m arterial PO_2_ is around 40 mmHg and S_a_O_2_ around 70% leading to a systolic PAP around 34 mmHg with a normal HPV in non-acclimatized healthy mountaineers (Bärtsch et al., 1991). Susceptibility to HAPE is associated with an excessive increase of systolic pulmonary artery pressure (PAP) during 2–4 h of normobaric hypoxia (F_I_O_2_ of 0.12) at low altitude (Grünig et al., 2000) and during hypobaric hypoxia at high altitude (Oelz et al., 1989; Bärtsch et al., 1991). It is important to note that the abnormal rise of PAP precedes the onset of HAPE by at least 12–24 h at 4,559 m (Figure 3). Right heart catheterization at 4,559 m showed increased mean PAP and as assessed by the single occlusion technique, increased capillary pressure in HAPE susceptible individuals (HAPE-S), while wedge pressures were normal. Interestingly, HAPE-S subjects, who developed pulmonary edema during the exposure, had the highest PAP values and each had a capillary pressure above the edema threshold (≥20 mmHg) for lungs unaccustomed to such elevations (Figure 4). Lower nitric oxide (NO) production, a lower hypoxic ventilatory response resulting in greater alveolar hypoxia and less hypocapnia, higher sympathetic tone and slightly lower lung volumes might all contribute to the greater hypoxic pulmonary vasoconstriction (HPV) in HAPE-S as discussed elsewhere (Bärtsch et al., 2005; Swenson and Bärtsch, 2012; Luks et al., 2017).

There are several hypotheses explaining how abnormally increased PAP leads to pulmonary edema. The most prevalent is inhomogeneity of regional HPV, for which there is some experimental evidence (Hopkins et al., 2005; Dehnert et al., 2006). Inhomogeneity of HPV would lead to areas of lower and higher perfusion resulting in alveolar edema with the higher capillary pressures in the high flow and less vasoconstricted areas (Hultgren, 1978). Other hypotheses include leakage across weaker arterioles branching off at right angles from larger pulmonary arteries (Goetz et al., 1996) and/or hypoxic venoconstriction.

Since there are individuals that do not develop HAPE despite strong HPV and abnormally increased PAP at high altitude (Dehnert et al., 2015), additional pathophysiologic factors must contribute to HAPE. Hypoxia induced inflammation and impaired alveolar fluid clearance have been considered and evaluated as possible contributors, but the few studies published have yielded inconclusive results regarding fluid clearance and evidence for inflammation.

**FIGURE 5:**
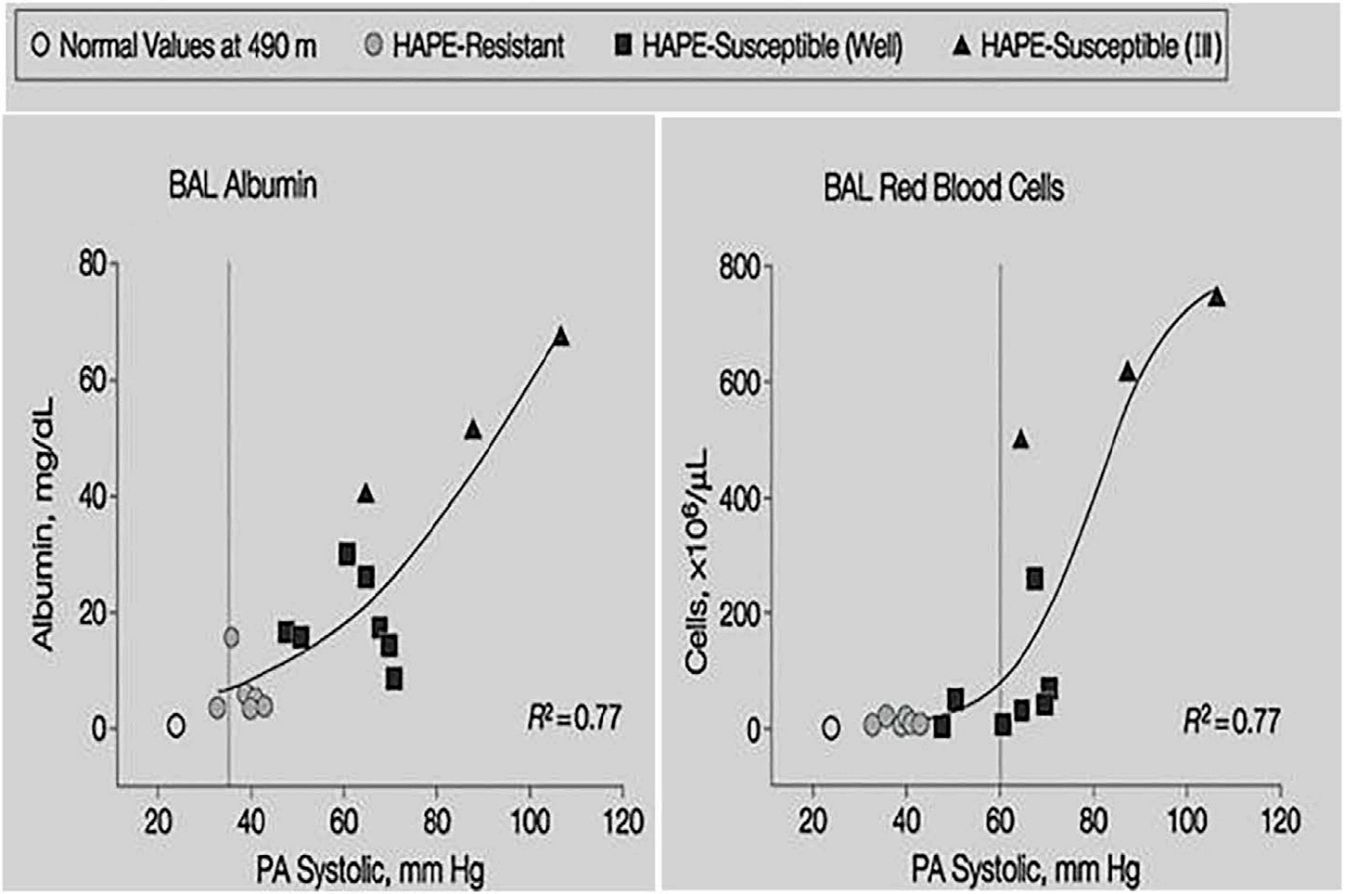
Individual BAL red cell count and albumin concentration plotted against systolic pulmonary artery pressure at 4,559 m. Shaded circles indicate values of HAPE resistant controls, filled squares indicate HAPE-susceptible subjects without HAPE and filled triangles indicate HAPE-susceptible subjects with HAPE. Data taken from Swenson, E.R., Maggiorini, M., Mongovin, S., Gibbs, J.S., Greve, I., Mairbaurl, H., Bartsch, P. 2002. Pathogenesis of high-altitude pulmonary edema: inflammation is not an etiologic factor. Jama, 287, 2228–35.

Broncho-alveolar lavage (BAL) in early HAPE at 4,559 m showed mild alveolar hemorrhage and increased albumin but no increase of neutrophils and pro-inflammatory cytokines (Swenson et al., 2002). Red cell count and albumin concentration in the BAL fluid correlated with the magnitude of pulmonary hypertension (Figure 5). Thus, early HAPE is a hydrostatic, non-cardiogenic edema and noninflammatory pulmonary edema. However, BAL in advanced HAPE of several days duration showed signs of inflammation in some but not all cases (Schoene et al., 1986; Kubo et al., 1997) suggesting that inflammation may occur as a secondary response to edema from physical disruption of the alveolar-capillary barrier (stress failure) and subsequent mild hemorrhage.

There is some indirect evidence that alveolar fluid clearance might be more impaired in HAPE-S subjects (Mairbaurl et al., 2003), e.g. reduced active nasal epithelium sodium absorption (Sartori et al., 2004), but interventions in mountaineers enhancing transepithelial alveolar fluid reabsorption are not conclusive because of multiple actions of salmeterol (Sartori et al., 2002) and dexamethasone (Maggiorini et al., 2006) as discussed in a review on HAPE in Comprehensive Physiology (Swenson and Bärtsch, 2012).

There are no studies investigating the effect of slow ascent in HAPE-S subjects. Anecdotal reports suggest, however, that HAPE in susceptible individuals ascending at an average rate of 350–400 m per day above 2,500 m remain well without medication up to at least 6,000 m. If such a slow ascent or a staged ascent are not possible, 30 mg bid of nifedipine sustained release or 10 mg bid of tadalafil should be recommended for a person with a history of HAPE. Lowering pulmonary artery pressure is the principal goal in treatment. This can be achieved by supplemental oxygen, portable hyperbaric bags and/or descent. If this is not possible, nifedipine or tadalafil should be given at the same dose as for prevention with the goal to use the clinical improvement to permit a safer descent (Bärtsch and Swenson, 2013).

### Comparison between IPE and HAPE

There are many similarities between IPE and HAPE, e.g., the occurrence in young, healthy, and fit individuals, clinical features such as hemoptysis, similar radiographic appearance (Lindholm et al., 2018), involvement of exercise, recurrence in susceptible individuals, and rapid recovery in a normal environment. There are, however, also considerable differences regarding the time course and the cardio-pulmonary mechanisms involved. IPE occurs within 1–2 h after immersion while HAPE develops more slowly over one to several days at high altitude suggesting that different hemodynamic responses and underlying mechanisms may lead to a similar type of a hydrostatic pulmonary edema.

#### Pulmonary circulation

Right heart catheterization shows the different starting points. Increased HPV leads to abnormally high PAP that precedes HAPE over several hours to days while PAWP remains normal at about 10 mmHg. Long intervals of continuous exercise at usually moderate levels is often undertaken and likely contributes to HAPE. HAPE, however, can also occur even after passive ascent at rest. It was shown that HPV is more regionally inhomogeneous in HAPE susceptible individuals and may lead to low and high flow areas with edema occurring in the latter (Hopkins et al., 2005; Dehnert et al., 2006).

Immersion in water of 20°C causes an immediate increase of PAWP of on average 18 mmHg - the critical threshold for edema formation - in IPE susceptible individuals (Moon et al., 2016), while non susceptible controls had on average only a rise to 13 mmHg. Low water temperature, higher sympathetic tone, and exercise may aggravate the increase in PAWP. Furthermore, the augmented preload with immersion may cause a slight increase in wall stiffness and impairment of diastolic function (Moon et al., 2016), particularly in older individuals or those with a history of systemic hypertension.

Mean PAP is also more increased in IPE prone individuals than IPE resistant controls after immersion, but the magnitude of this increase is considerably less than in patients with HAPE. It is likely that a greater part of the increase is a consequence of the higher PAWP (Breitling et al., 2015) since the transpulmonary pressure gradient (mean PAP – PAWP) exceeds the threshold >12 mmHg indicating out of proportion pulmonary hypertension only during exercise by 5 mmHg (Naeije et al., 2013). Alveolar hypoxia can be excluded as a cause of increased PAP in these studies (Peacher et al., 2010). HPV might, however, contribute to an increased PAP secondarily when gas exchange is impaired by edema leading to areas of low alveolar PO_2_. Since IPE usually occurs in heavily exercising individuals, it is likely that a high cardiac output contributes to pulmonary hypertension and edema since prolonged intense exercise in well trained runners or cyclists can lead to mild pulmonary edema even under dry conditions (Hopkins et al., 1997; Hodges et al., 2006; Zavorsky, 2007). Interestingly, heterogeneity of regional lung perfusion increases with sustained strenuous exercise (Burnham et al., 2009), which may explain similar distribution of edema as in HAPE and lowering the transpulmonary pressure gradient during exercise with sildenafil.

More severe hypoxemia and thus HPV may, however, play a role in IPE in breath-hold divers. The contribution of pulmonary hypertension to developing pulmonary edema in these conditions may, however, be small considering the short duration and other more important contributing factors such as negative intrapulmonary pressures and the cardiac effects of blood volume shifts on pulmonary capillary pressures.

Lowering pulmonary artery pressure in HAPE-S subjects by nifedipine (Bärtsch et al., 1991) or phosphodiesterase-5 inhibitors (Maggiorini et al., 2006) prevents HAPE and pulmonary vasodilators are also effective for treatment (Oelz et al., 1989). There are anecdotal reports that the posphoesterase-5 inhibitor sildenafil can prevent IPE in susceptible individuals, and it was shown that sildenafil lowers PAP but not PAWP significantly in submersed subjects with a history of SIPE exercising for 6 min (Moon et al., 2016). The effects of sildenafil on IPE could not be evaluated because of the short duration of such a demanding invasive study. The fact that sildenafil did not lower PAWP begs the question of whether the prevention of IPE with this drug is possible unless more than 6 min are needed for the changes in systemic venous capacitance to alter PAWP by a vasodilating action in the systemic circulation. Clearly, these concerns could be resolved by a placebo controlled real life study in swimmers with a history of IPE.

HAPE susceptible individuals have an abnormal increase of systolic PAP not only after a 2 h lasting exposure to hypoxia (F_I_O_2_ = 0.12) to above 40 mmHg but also during submaximal exercise in normoxia (Grünig et al., 2000). Thus, the question arises, whether IPE susceptible individuals have a similar reactivity of their pulmonary circulation to exercise (and possibly also to hypoxia) that could explain the rise in PAP during exercise while submersed. While we are not aware of such measurements during exercise in normoxia, an investigation in hypoxia (F_I_O_2_ = 0.12) did not find differences between IPE susceptible and control subjects (Ludwig et al., 2006), but the data are not conclusive, since the exposure lasted only 20 min. It would also be interesting to examine whether IPE susceptible individuals have an exaggerated HPV and could potentially be susceptible to HAPE, and if so, whether HAPE susceptible individuals might also be prone to IPE.

#### Type of alveolar capillary leak

BAL in HAPE shows large proteins like albumin and erythrocytes, the amount of which increase with higher PAP (Figure 3), and no markers of inflammation pointing to a pressure induced leak. The pressure necessary for rupture of the alveolo-capillary barrier were established in isolated perfused lung models (West et al., 1991), shown to occur *in vivo* in rats exposed to 9,000 m in 30 min (West et al., 1995) and the leak observed was named alveolar-capillary stress failure. Whether pressure induced breaks of the alveolo-capillary barrier as observed in the rats (West et al., 1995) are necessary to explain extravasation of high molecular weight proteins in HAPE has been questioned (Swenson et al., 2002; Kuebler, 2016). There is experimental evidence for a non-inflammatory increase of the permeability of the alveolo-capillary barrier such as transcellular passage through vesicular channels that form with high intracapillary pressures (Neal and Michel, 1996) or by a pressure induced activation of the endothelial contractile machinery (Yin et al., 2008). These changes occur without evident injury and are rapidly reversible.

While pink frothy sputum occurs predominantly in advanced stages of HAPE, hemoptysis has been reported frequently in individuals with IPE when associated with diving (Pons et al., 1995; Boussuges et al., 1999; Slade et al., 2001). It was shown that hemoptysis after breath-hold dives originates from areas below the vocal cords, but whether is bronchial bleeding or alveolar hemorrhage was not ascertained (Lindholm et al., 2008; Barković et al., 2021). Distal parenchymal bleeding could result from a pressure differential across the alveolar membrane or in consequence of mechanical shearing forces caused by compression. In scuba-divers, enhanced work of breathing adds to immersion and strenuous exercise with capillary damage resulting from excessive transcapillary pressure. There are preliminary data of a study reporting red blood cells and high molecular weight protein but no inflammatory markers in acute IPE (Ludwig et al., 2006) indicating that BAL fluid of hydrostatic edema with increased PAWP in IPE is similar to BAL in early HAPE.

## Conclusion

HAPE and IPE are non-inflammatory hydrostatic pulmonary edemas caused by different mechanisms. While exaggerated inhomogeneous HPV leading to increased capillary pressure due to overperfusion in the presence of normal wedge pressure is the major factor accounting for HAPE, increased pulmonary wedge and arterial pressures due to centralization of blood volume with immersion is the major cause of IPE. An increase of pulmonary arterial pressure due to increased cardiac output with exercise and responsiveness to pulmonary vasodilators may be an additional pathophysiologic factor leading to IPE, as in HAPE as well.
